# Prognostic significance of Naples prognostic score in operable renal cell carcinoma

**DOI:** 10.3389/fsurg.2022.969798

**Published:** 2022-09-27

**Authors:** Yaohui Wang, Xu Hu, Danxi Zheng, Yanxiang Shao, Thongher Lia, Xiang Li

**Affiliations:** ^1^ Department of Urology, West China Hospital of Sichuan University, Chengdu, China; ^2^ Department of Gynecology and Obstetrics, West China Second University Hospital, Sichuan University, Chengdu, China

**Keywords:** Naples prognostic score, renal cell carcinoma, neutrophil-to-lymphocyte ratio, lymphocyte-to-monocyte ratio, prognosis

## Abstract

**Background:**

Naples prognostic score (NPS), a novel scoring system based on nutritional and inflammatory status, is associated with prognosis in several cancers. This study aimed to evaluate the prognostic significance of preoperative NPS in patients undergoing nephrectomy.

**Patients and Methods:**

This study retrospectively analyzed patients with renal cell carcinoma (RCC) who underwent radical or partial nephrectomy between 2010 and 2013. The clinicopathological characteristics of patients stratified by preoperative NPS were compared. Survival analysis was performed using the Kaplan–Meier method and log-rank test. Univariate and multivariate Cox proportional hazards models were used to identify independent prognostic factors. Receiver operating characteristic curves were used to evaluate prediction efficiency.

**Results:**

A total of 638 patients with operable RCC were included. The high-NPS group (NPS group 2) was significantly associated with older age (*P* < 0.001), larger tumor size (*P* < 0.001), worse pathological T stage (*P* < 0.001), positive lymph node pathology (*P* = 0.002), higher tumor grade (*P* < 0.001), and greater tumor necrosis (*P* < 0.001). Multivariable analysis demonstrated that the high-NPS subgroup had significantly worse overall survival (OS) [hazard ratio (HR): 2.25, 95% confidence interval (CI): 1.45–3.50, *P* < 0.001] and progression-free survival (PFS) (HR: 2.26, 95% CI: 1.48–3.44, *P* < 0.001). Among several preoperative scoring systems, NPS had the strongest discriminatory power for predicting OS and PFS.

**Conclusion:**

Preoperative NPS can serve as a simple novel risk stratification tool to optimize the prognosis of patients with operable RCC. Further prospective and large-scale studies are needed to validate our findings.

## Introduction

The incidence of renal cell carcinoma (RCC) has increased recently, particularly in young populations (1, 2). In addition, it has been statistically reported that RCC caused 179,000 deaths globally in 2020, placing a heavy burden on social health (2). Currently, surgical nephrectomy has provided curative benefits for treating localized renal masses (3). Approximately 30% of patients, however, relapse or progress after surgery, and some may ultimately die (3–6). Therefore, it is beneficial to identify profitable prognostic indicators that guide patient risk stratification and surgical benefits.

Traditional prognostic systems mainly comprise the tumor–node–metastasis (TNM) stage and other histological evidence (3, 6). However, histological assessment often depends on the specimen after surgery; the TNM stage estimates the disease's tumor burden, ignoring information about host-related factors. Numerous studies have reported that host nutritional status and immune response are essential in the invasion and metastasis of most tumors (7, 8). Various peripheral blood markers, including neutrophil-to-lymphocyte ratio (NLR), lymphocyte-to-monocyte ratio (LMR), and platelet-to-lymphocyte ratio, have been validated to be significantly associated with the survival outcome in several kinds of cancer (9–12). Previous studies have reported that many nutrition-immune score systems incorporating the markers mentioned above, including prognostic nutritional index (PNI) and controlling nutritional status (CONUT), predict the prognosis of tumor patients (13–15).

A novel scoring system, the Naples prognostic score (NPS), based on NLR, LMR, serum albumin (sALB) level, and total cholesterol (TC) level, was first proposed by Galizia et al. (16). Reportedly, preoperative NPS is an independent prognostic factor in metastatic colorectal, pancreatic, lung, and endometrial cancers (17–20). However, its prognostic value in RCC remains unclear. Therefore, this study aimed to assess whether NPS can predict the prognosis of patients with RCC after nephrectomy.

## Patients and methods

### Patient selection

**Figure 1:**
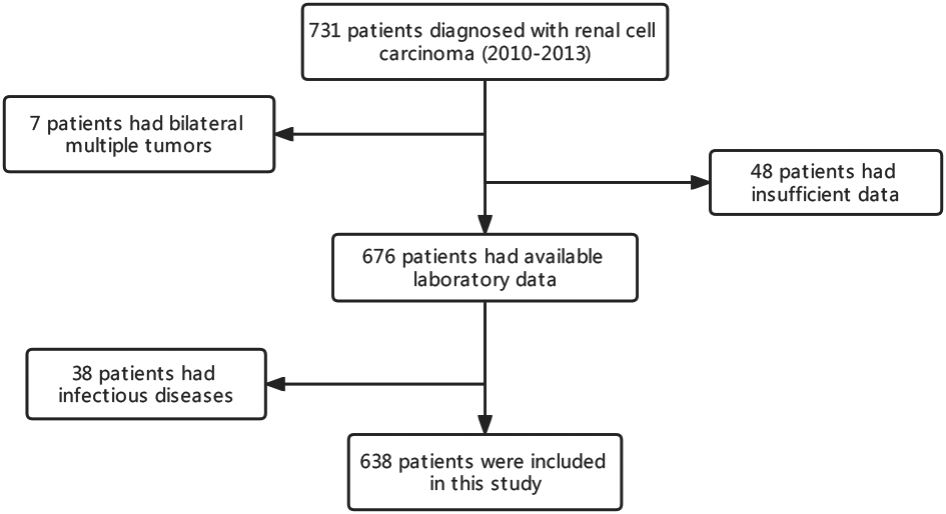
Flow diagram of the selection process.

A total of 731 patients diagnosed with RCC who underwent nephrectomy at Sichuan University West China Hospital between 2010 and 2013 were retrospectively assessed. Of these patients, those with multiple bilateral tumors (*n* = 7) and insufficient data (*n* = 48) were excluded. Furthermore, 38 patients with inflammatory diseases affecting their immunological status were excluded from the study. Finally, 638 patients were included for analysis (Figure 1). All patients underwent regular follow-ups and were examined for recurrence using laboratory tests, chest x-ray or computed tomography (CT) and ultrasound, or CT or magnetic resonance imaging of the abdomen, every 3 months for the first 2 years, every half a year for the next 3 years, and then once a year.

### Data collection

The following clinicopathological characteristics were obtained from the electronic medical records of the hospital: age, sex, smoking and drinking habits, laboratory tests, operation approach and type, histology, tumor size, T/N stage, grade, tumor necrosis, and sarcomatoid differentiation feature. Moreover, the absolute neutrophil, lymphocyte, and monocyte count, as well as serum albumin and total cholesterol levels, were all retracted within 7 days postoperatively. Tumor size was considered the longest length based on the pathological records. The pathological T/N stage was based on the eight edition of the TNM classification (21). The pathological assessment was based on the 2016 World Health Organization classification (22).

As previously reported by Galizia et al. (16), NPS took the dichotomous NLR values (if NLR > 2.96, point 1; if not, point 0), LMR (if LMR > 4.44, point 0; if not, point 1), serum albumin concentration (if albumin concentration <4.0 or 40 g/L, point 1; if not, point 0), and total cholesterol concentration (if cholesterol level >180 mg/ml or 4.65 mmol/L, point 0; if not, point 1) into consideration. The final NPS was calculated by summing the points mentioned above. NPS group 0 represented patients with an NPS of 0; NPS group 1 included those with NPS of 1–2; NPS group 2 included those with NPS of 3–4.

The clinical endpoints were overall survival (OS) and progression-free survival (PFS). OS was defined as the time from the date of the patient's operation to the last follow-up or all-cause death. PFS was defined as the time between surgery and the last follow-up or progression of the disease, while progression was defined as occurrence of new tumor lesions or at least a 20% increase in the sum of diameters of target lesions by imaging examination regularly, or even death.

### Statistical analysis

Student's *t*-test was employed to compare continuous variables [described as median with interquartile range (IQR)]; the chi-square and Fisher's exact tests were used to show the difference between categorized variables (presented as percentage frequency). Survival analyses of OS and PFS between the different subgroups were performed using Kaplan–Meier survival curves and the log-rank test. Time-dependent receiver operating characteristic (ROC) curves were used to show several parameters’ discriminatory power for predicting patient prognosis, including NPS, CONUT score, and PNI. Furthermore, the area under the curve (AUC) was calculated and compared.

Univariate and multivariate Cox proportional hazards regression models were used to determine independent prognostic factors of OS and PFS. Hazard ratios (HRs) with 95% confidence intervals (95% CIs) were used to demonstrate prognostic results. All statistical analyses were conducted using the R software version 4.0.3 (http://www.r-project.org/). A two-sided *P-*value <0.05 was regarded as a threshold for statistical significance.

## Results

### Clinicopathological characteristics of patients

A total of 638 patients with RCC were included in this study. The baseline patient clinicopathological characteristics are summarized in Table 1. The median age of the cohort was 55.5 years (IQR: 45.0–64.0). Of the patients, 394 were male (ratio = 61.8%) and 244 were female (ratio = 38.2%), with a median tumor size of 4.70 cm (IQR: 3.20–6.00). Concerning bad addiction, 175 (27.4%) and 127 (19.9%) patients were smokers and drinkers, respectively. Approximately two-third of the patients underwent open surgery (70.5%) and radical nephrectomy (RN) (68.8%). In addition, most (*n* = 539, 84.5%) patients had clear cell RCC. In addition, most patients had relatively better pathological T (T1–2, ratio = 87.9%) and N stages (N0, ratio = 98.0%). In contrast, a narrow patient had terrible tumor necrosis status (*n* = 77, ratio = 12.1%) and sarcomatoid differential features (*n* = 7, ratio = 1.10%). The median follow-up was 83.0 months (IQR: 74.0–92.8 months).

**Table 1:** Clinical characteristics of the patients between the NPS groups 0 and 1 and the NPS group 2.

	Total	NPS group		
		0 and 1	2	*P*-value
No. patients	638	529	109	
Age, median (IQR) (years)	55.5 (45.0–64.0)	54.0 (45.0–63.0)	61.0 (49.0–68.0)	<0.001
Gender				0.008
Male	394 (61.8%)	314 (59.4%)	80 (73.4%)	
Female	244 (38.2%)	215 (40.6%)	29 (26.6%)	
Smoking				1.000
+	175 (27.4%)	145 (27.4%)	30 (27.5%)	
−	463 (72.6%)	384 (72.6%)	79 (72.5%)	
Drinking				0.635
+	127 (19.9%)	103 (19.5%)	24 (22.0%)	
−	511 (80.1%)	426 (80.5%)	85 (78.0%)	
Operative approach				0.195
Open	450 (70.5%)	367 (69.4%)	83 (76.1%)	
Laparoscopic	188 (29.5%)	162 (30.6%)	26 (23.9%)	
Nephrectomy				0.005
Radical	439 (68.8%)	351 (66.4%)	88 (80.7%)	
Partial	199 (31.2%)	178 (33.6%)	21 (19.3%)	
Tumor size, median (IQR) (cm)	4.70 (3.20–6.00)	4.50 (3.10–5.60)	6.00 (4.00–9.80)	<0.001
Histological subtype				0.200
Clear cell	539 (84.5%)	442 (83.6%)	97 (89.0%)	
Non-clear cell	99 (15.5%)	87 (16.4%)	12 (11.0%)	
Pathological T stage				<0.001
T1–2	561 (87.9%)	485 (91.7%)	76 (69.7%)	
T3–4	77 (12.1%)	44 (8.32%)	33 (30.3%)	
Pathological N stage				0.002
N0	625 (98.0%)	523 (98.9%)	102 (93.6%)	
N1	13 (2.04%)	6 (1.13%)	7 (6.42%)	
Tumor grade				<0.001
G1–2	346 (54.2%)	315 (59.5%)	31 (28.4%)	
G3–4	292 (45.8%)	214 (40.5%)	78 (71.6%)	
Tumor necrosis				<0.001
+	77 (12.1%)	45 (8.51%)	32 (29.4%)	
−	561 (87.9%)	484 (91.5%)	77 (70.6%)	
Sarcomatoid differentiations				0.342
+	7 (1.10%)	5 (0.95%)	2 (1.83%)	
−	631 (98.9%)	524 (99.1%)	107 (98.2%)	
NLR, median (IQR)	2.17 (1.67–2.84)	1.99 (1.60–2.48)	3.47 (3.07–4.37)	<0.001
LMR, median (IQR)	5.15 (3.74–6.64)	5.60 (4.48–7.13)	3.13 (2.47–3.58)	<0.001
sALB, median (IQR) (g/L)	42.8 (40.3–45.0)	43.2 (41.2–45.3)	39.0 (36.1–41.9)	<0.001
TC, median(IQR) (mmol/L)	4.39 (3.86–5.03)	4.61 (4.02–5.20)	3.86 (3.27–4.27)	<0.001
Naples prognostic score				<0.001
0	144 (22.6%)	144 (27.2%)	0 (0.00%)	
1	235 (36.8%)	235 (44.4%)	0 (0.00%)	
2	150 (23.5%)	150 (28.4%)	0 (0.00%)	
3	68 (10.7%)	0 (0.00%)	68 (62.4%)	
4	41 (6.43%)	0 (0.00%)	41 (37.6%)	

NPS, Naples prognostic score; IQR, interquartile range; NLR, neutrophil-to-lymphocyte ratio; LMR, lymphocyte-to-monocyte ratio; sALB, serum albumin; TC, total cholesterol.

Based on the NPS system, the subgroups were as follows: NPS groups 0–1, 529 cases; NPS group 2, 109 patients. Statistical analysis showed that patients in NPS group 2 (high-NPS) were significantly associated with older age (*P* < 0.001), larger tumor size (*P* < 0.001), worse pathological T stage (*P* < 0.001), positive lymph node pathology (*P* = 0.002), higher tumor grade (*P* < 0.001), and greater tumor necrosis (*P* < 0.001) than those in the other subgroups.

### Survival analysis based on NPS

**Figure 2:**
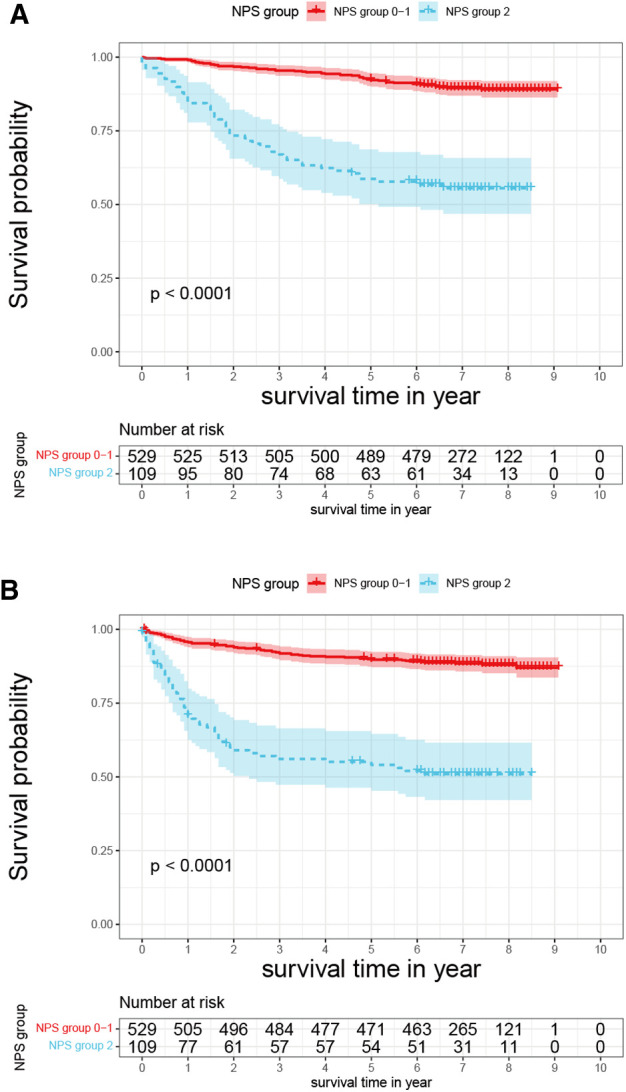
Kaplan–Meier curves of (**A**) overall survival and (**B**) progression-free survival for NPS groups 0 and 1 and group 2. NPS, Naples prognostic score.

Kaplan–Meier OS and PFS curves for the NPS subgroups are shown in Figure 2. A significant statistical difference in survival was observed between the NPS subgroups (OS: *P* < 0.001, Figure 2A; PFS: *P* < 0.001, Figure 2B). Lower NPS was significantly associated with higher OS and PFS benefits. In the low- and high-NPS subgroups, the 5-year OS rates were 92.63% and 57.80%, and the 5-year PFS rates were 89.04% and 49.54%, respectively.

Univariate and multivariate Cox regression analyses identified NPS as an independent prognostic factor for OS and PFS. The high-NPS subgroup showed significantly worse OS outcomes (HR: 2.25, 95% CI: 1.45–3.50, *P* < 0.001) and shorter PFS (HR: 2.26, 95% CI: 1.48–3.44, *P* < 0.001). The following factors were considered independent prognostic factors for OS: age, surgical approach, operation type, tumor size, pathological T and N stage, tumor grade, tumor necrosis, and sarcomatoid differentiation (Table 2).

**Table 2:** Univariate and multivariate analysis of prognostic factors for OS and PFS in the patients with RCC (*n* = 638).

	OS				PFS			
	Univariate		Multivariate		Univariate		Multivariate	
	HR (95% CI)	*P*-value	HR (95% CI)	*P*-value	HR (95% CI)	*P*-value	HR (95% CI)	*P*-value
Age (per unit increase)	1.04 (1.02–1.05)	<0.001	1.02 (1.01, 1.04)	0.007	1.02 (1.01–1.04)	<0.001	1.02 (1.00, 1.03)	0.068
Gender (male vs. female)	1.36 (0.90–2.06)	0.142			1.58 (1.05–2.37)	0.027	0.94 (0.61, 1.45)	0.776
Smoking (+ vs. −)	1.16 (0.76–1.77)	0.492			1.13 (0.75–1.69)	0.561		
Drinking (+ vs. −)	0.89 (0.54–1.47)	0.661			0.96 (0.60–1.53)	0.871		
Operative approach (laparoscopic vs. open)	0.21 (0.11–0.41)	<0.001	0.24 (0.12, 0.48)	<0.001	0.31 (0.18–0.54)	<0.001	0.52 (0.29, 0.94)	0.030
Nephrectomy (partial vs. radical)	0.14 (0.07–0.31)	<0.001	0.35 (0.16, 0.79)	0.011	0.13 (0.06–0.27)	<0.001	0.34 (0.15, 0.76)	0.008
Tumor size (per unit increase)	1.30 (1.24–1.36)	<0.001	1.06 (1.01, 1.13)	0.029	1.32 (1.27–1.38)	<0.001	1.09 (1.03, 1.15)	0.004
Histology subtype (non-clear cell vs. clear cell)	1.01 (0.59–1.72)	0.97			0.90 (0.53–1.52)	0.689		
Pathological T stage (T3–4 vs. T1–2)	8.58 (5.8–12.69)	<0.001	2.69 (1.71, 4.22)	<0.001	9.62 (6.61–14)	<0.001	3.11 (2.02, 4.79)	<0.001
Pathological N stage (N1 vs. N0)	16.3 (8.91–29.82)	<0.001	7.08 (3.52, 14.23)	<0.001	18.3 (9.98–33.58)	<0.001	7.90 (3.93, 15.91)	<0.001
Tumor grade (G3–4 vs. G1–2)	5.40 (3.34–8.72)	<0.001	2.36 (1.41, 3.93)	0.001	7.12 (4.34–11.66)	<0.001	3.30 (1.96, 5.56)	<0.001
Tumor necrosis (+ vs. −)	4.84 (3.23–7.28)	<0.001	1.65 (1.06, 2.59)	0.027	5.4 (3.67–7.96)	<0.001	1.77 (1.16, 2.71)	0.008
Sarcomatoid differentiations (+ vs. −)	5.42 (1.99–14.75)	<0.001	3.81 (1.36, 10.71)	0.011	4.99 (1.84–13.56)	0.002	3.64 (1.30, 10.19)	0.014
NPS group (NPS group 2 vs. NPS group 0–1)	5.66 (3.83–8.33)	<0.001	2.25 (1.45, 3.50)	<0.001	5.68 (3.91–8.23)	<0.001	2.26 (1.48, 3.44)	<0.001

HR, hazard ratio; 95% CI, 95% confidence interval; NPS, Naples prognostic score; OS, overall survival; PFS, progression-free survival; RCC, renal cell carcinoma.

### Discriminatory strength of three preoperative scoring system

**Figure 3:**
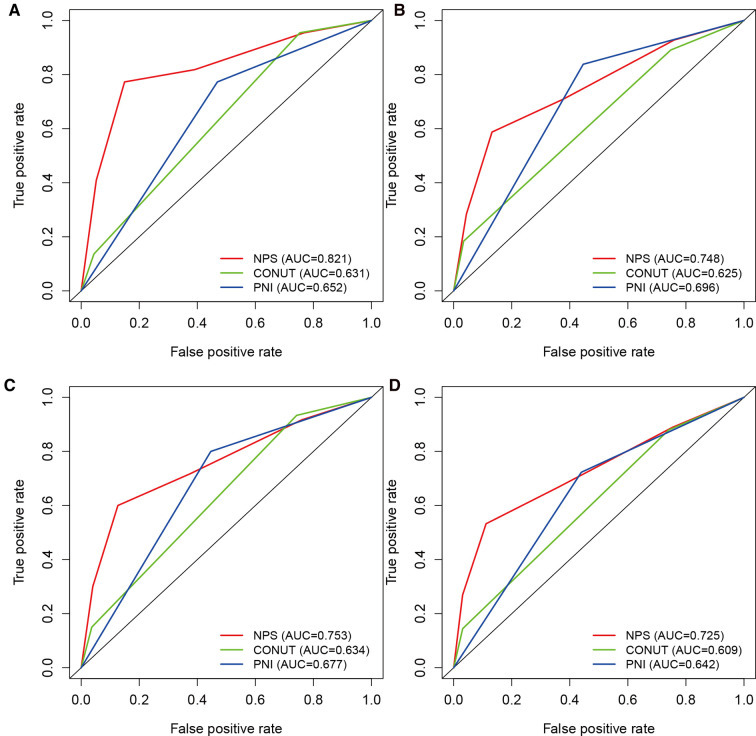
ROC curves revealing the discriminatory power of NPS, CONUT, and PNI indexes for predicting (**A**) 1-year OS, (**B**) 1-year PFS, (**C**) 3-year OS, and (**D**) 3-year PFS. OS, overall survival; PFS, progression-free survival; NPS, Naples prognostic score; CONUT, controlling nutritional status; PNI, prognostic nutritional index.

Time-dependent ROC curves revealing the discriminatory power of the three scoring systems are shown in Figure 3. The AUC values of NPS for predicting 1-year OS and PFS were 0.821 and 0.748, respectively (Figures 3A,B). The NPS indicator had a significantly stronger AUC value than the CONUT and PNI indicators. Similarly, NPS had the largest AUC for predicting 3-year OS and PFS (OS: 0.753, Figure 3C; PFS: 0.725, Figure 3D) compared with CONUT and PNI. Therefore, NPS had the strongest discriminatory power for predicting OS and PFS among the three preoperative scoring systems.

## Discussion

In the present study, we assessed the prognostic value of NPS in patients with RCC. Correlated with acknowledged adverse factors, including older age, tumor size, pathological T/N stage, tumor grade, and tumor necrosis histological features, the high-NPS group predicted worse OS and PFS survival outcomes. Univariate and multivariate analyses showed that NPS was an independent prognostic factor for OS and PFS.

Interestingly, although the median tumor size was 4.70 cm, the majority of patients underwent RN and not partial nephrectomy (PN), which could be the reason that many research studies, such as EORTC (23), did not support a survival benefit to PN, making RN more between 2010 and 2013. However, it did not seem to affect our conclusion about prognostic significance of NPS after using proper statistical approaches to minimize the influence of operation types.

NPS, a novel inflammation-related prognostic score reported first in 2017 (16), comprehensively considers NLR, LMR, and serum albumin and total cholesterol levels. NLR and LMR include three types of inflammation-related cells: neutrophils, lymphocytes, and monocytes. Neutrophils can secrete substances, including vascular endothelial growth factor and several soluble neutrophil granules, inducing angiogenesis and tumor cell proliferation (24), remodeling the matrix, and interfering with T-cell-dependent antitumor immunity (25–27). The interaction between neutrophils and circulating tumor cells facilitates tumor cell binding to the endothelium during tumor metastasis (26). A low absolute neutrophil count in lung cancer indicates improved survival after immunotherapy (28). Lymphocytes play a fundamental role in adaptive immune responses, induce cytotoxic immune responses, and participate in the tumor microenvironment (TME). Tumor-infiltrating lymphocytes play a role in immunosurveillance (29) and regulate tumor progression and migration in many tumors, including melanoma, lung cancer, and RCC (30–32). Hence, a few lymphocytes showed mild antitumor immunological activity. Many studies have reported that lymphopenia accounts for poor survival in patients with gastric cancer and papillary RCC (33, 34). Monocytes are considered the initiators of innate immune responses. They also play a vital part in TME, namely, tumor-associated macrophages (TAMs). Currently, TAMs (divided into antitumor M1-like and pro-tumor M2-like TAMs) are regarded as essential drivers of tumor progression, metastasis, and drug resistance (35). M2-polarized TAMs have been reported to enhance angiogenesis and tumor growth by targeting many molecules, including interleukins (36, 37). Consequently, the prognostic mechanisms of NLR and LMR are easily understood, considering the above-mentioned theories. Therefore, higher NLR and lower LMR in NPS group 2 might indicate a greater tumor burden, resulting in higher pathological T and N stages, higher grades, and more significant tumor necrosis in NPS group 2.

In addition, two additional parameters, serum albumin and TC levels, were included in the NPS. Albumin, the most abundant protein in the human blood, plays a vital role in transporting compounds and stimulating tissue repair (38, 39). As a result, older people with poorer nutritional status were more likely to be categorized in the NPS group 2. Furthermore, cytokines and other proinflammatory substances reduced albumin concentration (40). Therefore, a decreased albumin level reflects malnutrition status and the intensity of the inflammatory response (41). Recently, many studies have found that sALB is associated with the prognosis of patients with RCC and worse OS, cancer-specific survival, recurrence-free survival, and PFS in the population with a lower preoperative sALB level (42–44). However, albumin concentration is influenced by many factors, including changes in body fluid volume. Therefore, the total cholesterol level was adopted to further evaluate the nutritional status. Cholesterol, a basic component of the cell membrane, mediates cell surface receptors’ mobility, interfering with the transmission of transmembrane signals (45). Consequently, many immunocompetent cells lose their immune clearance function owing to decreased cholesterol levels in their cytomembranes. In recent decades, evidence has shown that lipid metabolism and cancer onset are highly correlated (46). These observations could lead to tumor cell escape from the host immune system, accounting for increased proportion of circulating tumor cells and facilitating tumor invasion and metastasis. Based on these observations, NPS may be a credible prognostic predictor in patients with cancer.

Besides the underlying biochemical mechanisms mentioned above, clinical studies have confirmed NPS indicators’ values. Li et al. found that preoperative status was an independent prognostic predictor of OS and PFS in grade 2/3 endometrial cancer (multivariate analysis: OS: HR: 4.066, 95% CI: 1.076–15.37, *P* = 0.039; PFS: HR: 6.752, 95% CI: 1.537–29.671, *P* = 0.011) (17). Nakagawa et al. also showed NPS’s value for predicting OS independently in patients with resected pancreatic cancer (HR: 1.82, 95% CI: 1.15–2.84) (20). Kano et al. demonstrated that NPS can predict prognosis in patients with locally advanced esophageal squamous cell carcinoma and is more reliable and accurate than other systemic inflammatory and nutritional indices (47). Chen et al. retrospectively analyzed 173 patients with HER2-positive breast cancer and identified that increased NPSs correlated significantly with poor OS and disease-free survival (both *P* < 0.001) (48). The present results are consistent with those of previous studies. Moreover, our study is the first to identify NPS's prognostic value in an operable RCC population.

Preoperative NPS may be beneficial in clinical practice, evaluating the host's nutrition and immune status more comprehensively than only one type of inflammatory cell or one nutrition-related biomarker. Unlike pathological results, the numerous NPS items were easy to obtain because a series of laboratory tests, including complete blood count and serum albumin and total cholesterol level determination, was performed before surgery. In the existing literature, there are several kinds of scoring systems for malignant tumors, including the modified Glasgow prognostic score (mGPS), CONUT, and PNI. However, preoperative C-reactive protein (CRP) levels had to be determined to calculate GPS. CRP tests are not routinely performed in patients with RCC, limiting the use of the mGPS in actual clinical diagnosis and treatment. Concerning the CONUT score and PNI, NPS performed better in predicting the prognosis of patients with RCC than these two indices.

Our study has some limitations. First, as a retrospective cohort study, this study inevitably had potential selection bias. Second, the present study, which lacked external validation, was a single-center cohort study, and the moderate sample size might account for the attenuation of demonstrative power. Therefore, a multiple-center, large-scale, prospective validation study is required. Third, some other unknown elements might have disturbed the neutrophil, lymphocyte, and monocyte counts, as well as the sALB and TC levels, despite excluding patients with inflammatory diseases. Finally, the NPS value was determined preoperatively based on laboratory tests at some time points. We focused on the relationship between preoperative NPS and prognosis; however, dynamic changes in NPS are also valuable. Therefore, a prospective validation analysis of NPS's dynamic prognostic role is necessary.

## Conclusion

NPS, a novel and simple scoring system, is an independent preoperative predictor of OS and PFS in patients with RCC who underwent surgery. The present research conclusions are similar to previous findings concerning metastatic colorectal cancer, pancreatic cancer, endometrial cancer, and early-stage non-small-cell lung cancer. This discovery highlights the significance of novel peripheral inflammatory biomarkers and outcomes in patients with RCC. Further large-scale prospective studies on NPS are essential to validate our findings.

## Data Availability

The raw data supporting the conclusions of this article will be made available by the authors, without undue reservation.
